# Communicating bronchopulmonary foregut malformations: Revisiting a rare entity

**DOI:** 10.4102/sajr.v30i1.3453

**Published:** 2026-05-18

**Authors:** Abhinav C. Bhagat, Radha Sarawagi, Allen Johnson, Rajesh Malik, Anita Mathew

**Affiliations:** 1 Department of Radiodiagnosis, All India Institute of Medical Sciences, Bhopal, India; 2 Department of Neuroimaging and Interventional Radiology, National Institute of Mental Health and Neurosciences, Bangalore, India

**Keywords:** communicating bronchopulmonary foregut malformation, oesophageal lung, oesophageal bronchus, tracheo-oesophageal fistula, fluoroscopy, computed tomography, VACTERL

## Abstract

Communicating bronchopulmonary foregut malformations (CBPFM) are rare developmental tracheo-bronchial anomalies characterised by a patent communication between the respiratory system and upper gastrointestinal tract. Clinical diagnosis is challenging because of non-specific symptoms and overlapping clinical presentation with tracheo-oesophageal fistula. Imaging plays a key role in early diagnosis and management of this rare entity. This case report describes two cases of CBPFM, highlighting the key imaging findings on various modalities along with a brief review of literature.

**Contribution:**

This case report underscores the typical imaging features of CBPFM and the role of imaging in differentiating it from the more common tracheo-oesophageal fistula.

## Introduction

Congenital bronchopulmonary foregut malformations are a diverse set of developmental tracheo-bronchial anomalies. These are often associated with anomalies of the upper gastrointestinal tract, pulmonary, and systemic arterial supply and venous drainage.^1^

A rare manifestation of these malformations presents as a patent congenital communication between the respiratory system (trachea, bronchus or lung) and upper gastrointestinal tract (oesophagus or stomach), referred to as communicating bronchopulmonary foregut malformations (CBPFM).^2^ These malformations may often go undetected because of non-specific clinical features, which may overlap with the more common tracheo-oesophageal fistula. This case report describes two cases along with a brief review of the literature and discussion of relevant embryology, highlighting the critical role of the radiologist in the diagnosis of this uncommon condition.

## Patient presentation

### Case 1

A 17-month-old girl presented with complaints of frequent episodes of fever, not associated with cough or upper respiratory tract infection. Examination on admission revealed decreased air entry on the right side of the chest and an umbilical hernia. There was a history of previous neonatal Intensive Care Unit (ICU) admission at 1 month of age and hospitalisation elsewhere at 4 months of age for possible lower respiratory tract infection. Pregnancy was full-term and uneventful with normal vaginal delivery and normal examination at birth.

The chest radiograph revealed opacification of the right hemithorax with ipsilateral mediastinal shift. Ultrasound of the abdomen demonstrated an umbilical hernia and inferior cross fused renal ectopia on the right side. Fluoroscopic contrast oesophagogram demonstrated a contrast-opacified bronchus arising from the distal oesophagus with its branches extending into the right hemithorax (Figure 1).

**FIGURE 1:**
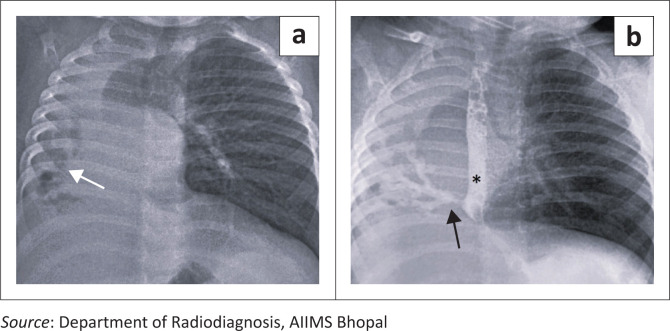
(a, b) Scout fluoroscopic image (a) of the chest reveals white-out of right hemithorax with an air bronchogram (white arrow) in the lower zone. Volume loss is evident in the form of ipsilateral tracheo-mediastinal shift and retrosternal herniation of the hyperexpanded left upper lobe. Fluoroscopic spot image after administration of oral positive contrast (b) shows passage of contrast from the distal oesophagus (asterisk) into the bronchial tree through a patent communication (black arrow).

Contrast-enhanced CT of the thorax and abdomen demonstrated complete collapse-consolidation of the right lung with ipsilateral mediastinal shift and compensatory hyperinflation of the left lung. Mildly dilated, aerated subsegmental bronchi were present. The trachea was seen continuing as the left main bronchus. The right main bronchus was not arising from the trachea. Instead, the right lung was supplied by an accessory bronchus arising from the distal oesophagus, approximately 2.8 cm proximal to the gastro-oesophageal (GO) junction. This accessory bronchus measured 6 mm in diameter and 12 mm in length before branching into smaller airways. Administration of oral contrast revealed passage of contrast from the oesophagus into this accessory bronchus and its branches (Figure 2). Arterial supply of the small right lung was derived from an attenuated right pulmonary artery (4.6 mm in maximum diameter compared to 10.5 mm on left side) with non-visualisation of its superior division (Figure 3), suggesting right pulmonary hypoplasia. The right lung was drained by the right inferior pulmonary vein with non-visualisation of the right superior pulmonary vein. No systemic arterial supply or venous drainage was identified.

**FIGURE 2:**
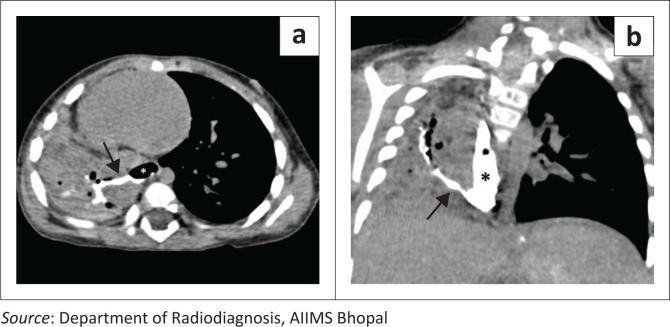
(a, b) Axial (a) and coronal oblique (b) reformatted mediastinal window CT images in the same patient reveal collapse-consolidation of the right lung which is supplied by an accessory bronchus (arrows) arising from the distal oesophagus (asterisk). The orally administered positive contrast material is seen passing freely from the oesophagus into the bronchial tree.

**FIGURE 3:**
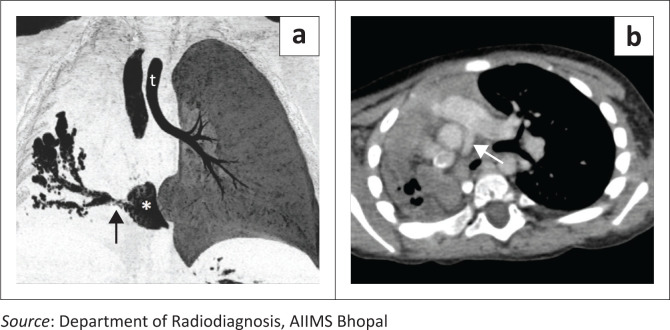
(a, b) Coronal minimum intensity projection (a) image shows the accessory bronchus (black arrow) supplying the right lung and arising from the distal oesophagus (asterisk). Bronchiectatic changes are also seen on the right side. The trachea (*t*) is seen continuing as the left main bronchus without giving off the right main bronchus. Axial (b) contrast-enhanced CT image demonstrates a hypoplastic right pulmonary artery (white arrow) supplying the affected right lung.

Associated anomalies included right-sided inferior cross-fused renal ectopia, umbilical hernia, intestinal malrotation, vertebral segmentation and fusion abnormalities, and supernumerary ribs. Echocardiography did not reveal any cardiac abnormalities. There were no associated limb anomalies.

The child underwent thoracotomy and right sided pneumonectomy with closure of the distal oesophageal communication. The child was well at the time of discharge following surgery and healthy on routine follow-up.

### Case 2

A 26-day-old male neonate presented with respiratory distress syndrome, pneumonia, sepsis and positive COVID-19 inflammatory markers. The neonate was placed on ventilatory support. Antenatal history was uneventful with full-term normal vaginal delivery and normal examination at birth.

The chest radiograph revealed consolidation of the left middle and lower zones. Contrast-enhanced CT of the thorax was performed, which revealed consolidation of the left lower lobe with air-bronchograms and cylindrical bronchiectasis. The airways supplying the left lower lobe were seen uniting into a common bronchus, which joined the distal oesophagus, approximately 1.4 cm proximal to the GO junction (Figure 4).

**FIGURE 4:**
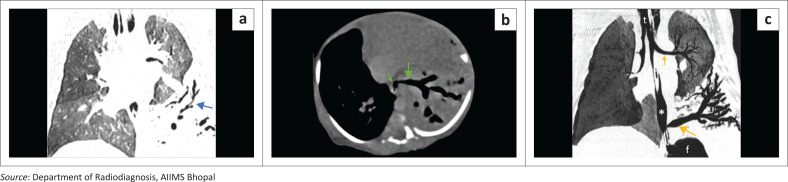
(a–c) Coronal (a) lung window CT image reveals consolidation with bronchiectatic changes (blue arrow) in the left lower lobe. Axial (b) mediastinal window CT image shows the bronchus (thick green arrow) supplying the left lower lobe and communicating with the distal oesophagus (thin green arrow). Coronal minimum intensity projection image (c) demonstrates the left lower lobe bronchus (thick yellow arrow) originating from distal oesophagus (asterisk), whereas the left upper lobe bronchus (thin yellow arrow) is seen arising from the trachea (*t*). Gaseous distension of gastric fundus (*f*) is observed.

This accessory bronchus measured 17 mm in length and 4 mm in maximum diameter. There was no communication between the dilated airways and left main bronchus. The left main bronchus and its branches were seen supplying the left upper lobe. The right main bronchus was arising from the trachea to supply the right lung. Arterial supply of the affected lobe was from a prominent artery arising from the coeliac axis (Figure 5), which measured 5.2 mm in maximum diameter. The left pulmonary artery and its branches were seen to supply only the left upper lobe and not the affected left lower lobe. Venous drainage of the affected left lower lobe was seen via prominent veins draining into a dilated left inferior pulmonary vein, which formed a common trunk with the left superior pulmonary vein and joined the left atrium (LA). Echocardiography showed an atrial septal defect measuring approximately 5 mm in width.

**FIGURE 5:**
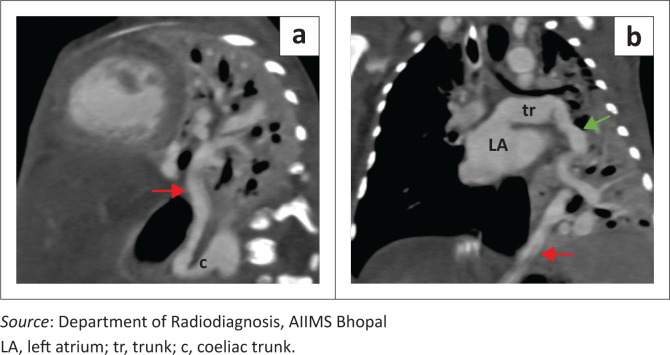
(a, b) Sagittal (a) and coronal oblique (b) reformatted contrast-enhanced CT images reveal a prominent artery (red arrows) arising from the coeliac trunk (c) supplying the left lower lobe. Venous drainage is seen via the inferior pulmonary vein (green arrow) draining into a common trunk (tr), which opens into the left atrium (LA).

No other associated anomalies were seen. Unfortunately, the neonate’s condition continued to deteriorate, and he demised 2 days later.

## Discussion and literature review

The term congenital bronchopulmonary foregut malformation encompasses a variety of congenital anomalies of the lung parenchyma, airways, oesophagus and foregut vascular anomalies (congenital pulmonary airway malformation, sequestration, duplication cysts, congenital lobar overinflation, bronchial atresia, pulmonary sling etc.) within its ambit.^3^ They are considered rare, with an estimated incidence of 30–42/100 000 individuals.^1^ These malformations are postulated to have a common embryological origin, and have been further classified as those without a gastrointestinal connection, those with a patent gastrointestinal connection and those with a non-patent gastrointestinal connection.^4^

Malformations with a patent gastrointestinal connection are commonly referred to as CBPFM. In 1992, Srikanth et al.^5^ reviewed 57 such cases and proposed a classification system for CBPFM (Figure 6) into the following four groups.

**FIGURE 6:**
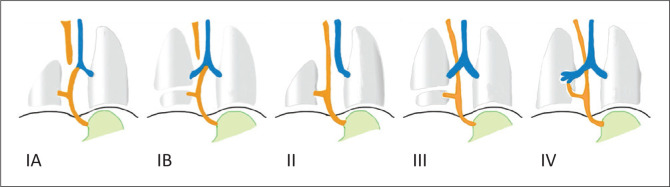
Schematic diagram depicting the classification of communicating bronchopulmonary foregut malformations.

Group I (16%): Associated with oesophageal atresia and tracheo-oesophageal fistula. It is further subdivided into group IA (completely sequestered lung showing communication with the foregut) and group IB (sequestered anatomic lobe or segment showing communication with the foregut).

Group II (33%): One lung originates from the lower oesophagus.

Group III (46%): An isolated anatomic lung lobe or segment communicates with the oesophagus or stomach.

Group IV (5%): A portion of the normal bronchial system communicates with the oesophagus. The portion of the lung served by the communicating bronchus receives systemic blood supply.

If the bronchus originates from the oesophagus and supplies the lung segment, it is referred to as oesophageal bronchus; while if the entire lung is supplied by a bronchus arising from the oesophagus, it is called oesophageal lung.

Congenital bronchopulmonary foregut malformations closely resemble pulmonary sequestration and have a similar embryological basis of development. Pulmonary sequestration arises when an additional supernumerary tracheo-bronchial bud develops from the primitive foregut distal to the normal tracheo-bronchial lung bud.^6^ If the accessory bud arises before the development of pleura, it is enveloped by the normally developing lung and forms an intralobar sequestration. If the accessory lung bud develops after the pleura has already formed, it grows separately from the normally developing lung in its own pleural covering and forms an extralobar sequestration.^7^ Both extra- and intralobar sequestrations show systemic arterial supply, which usually arises from the thoracic or abdominal aorta or its branches, with venous drainage being variable. Most extralobar sequestrations (80%) show systemic drainage through the azygos or hemiazygos system with the remainder showing venous drainage partly through pulmonary veins.^8^ Intralobar sequestrations drain via the pulmonary veins in the majority of cases (95%), with few cases showing systemic drainage through the azygos or hemiazygos system. Nevertheless, clearcut differentiation between the two types is not always possible owing to the mixed type of venous drainage to both the pulmonary and systemic circulations.^9^ Moreover, it has been postulated that intralobar sequestration may be an acquired lesion as a consequence of repeated infections resulting in chronic inflammation and recruitment of aberrant vessels from the aorta and its branches.^10^

Most cases of sequestration do not exhibit a patent foregut communication because of complete involution of the pedicle during embryogenesis. A small notch on the outer wall of the oesophagus at the site of prior embryonic communication can be seen in such cases.^7^ Intermediate stages of the involution process have been described in sequestrations where a remnant of the foregut communication is seen in the form of a fibrous-like pedicle that accompanies the main arterial supply of the bronchopulmonary mass. If the pedicle connecting the accessory bronchopulmonary tissue and the primitive foregut does not involute, the sequestration remains in free communication with the gastrointestinal tract.^7,11^

Clinically, most patients with CBPFM present in infancy and early childhood; late presentation in adulthood may be seen in rare cases with neonatal presentation even more rare.^12,13,14^ Prenatal diagnosis of oesophageal bronchus using ultrasound and MRI has also been described.^15^ Symptoms include chronic cough, respiratory distress exacerbated by feedings, and recurrent pneumonia.^16^ Associated vertebral, anal, cardiac, tracheo-oesophageal, renal and limb (VACTERL) abnormalities are also described and should be excluded by a comprehensive evaluation. Rarely, CBPFM can occur bilaterally.^17^

Imaging plays a crucial role in differentiating tracheo-oesophageal fistula from CBPFM, as both these conditions have overlapping clinical presentations. The chest radiograph is obtained as part of initial evaluation and may reveal consolidation or collapse of the affected lobe or lung segment with variable presence of air bronchograms and bronchiectasis.

Contrast oesophagogram is usually performed in cases with a strong clinical suspicion of an abnormal communication between the airway and oesophagus. It is performed using water-soluble iodinated non-ionic contrast, which achieves adequate distension of the oesophagus and demonstrates an abnormal communication between the proximal oesophageal segment and trachea in case of tracheo-oesophageal fistula. It can also detect type E (H-type) tracheo-oesophageal fistula; the feeding tube is inserted into the lower oesophagus and gradually withdrawn carefully during simultaneous injection of contrast under continuous fluoroscopic monitoring using cross-table lateral imaging with the child positioned in the prone or semi-prone position.^18^

Similarly, contrast oesophagogram can demonstrate an abnormal communication between the oesophagus and airways in CBPFM, except in group I because of the presence of oesophageal atresia. Group II–IV CBPFMs are well-demonstrated on contrast oesophagogram, which reveals an accessory bronchus (separate from the normal tracheo-bronchial tree) arising from the oesophagus and supplying the entire lung (group II), anatomical lobe or segment (group III) or communicating with the normal bronchial system (group IV) of the affected lung.^2^

Contrast-enhanced CT of the thorax with oral contrast administration can demonstrate the abnormal communication in greater detail and provides the advantage of multiplanar reformatting, which is useful for surgical planning. Importantly, it plays a crucial role in the evaluation of vascular structures. The abnormal lung tissue and its relation with surrounding structures, and the vascular supply and venous drainage of the affected lung or lung segment is well evaluated on cross-sectional imaging. Aberrant systemic arterial supply to the affected lung (esp. lower lobes) may arise from the infra-diaphragmatic aorta or its branches (such as the coeliac artery, as in the second case). Therefore, scan coverage should include the abdominal aorta up to the origin of renal arteries. CT is also useful in depicting associated arterial and venous anomalies such as anomalous pulmonary venous drainage, interrupted or hypoplastic pulmonary artery, pulmonary artery sling and azygous continuation of the inferior vena cava.^19^ Associated vertebral and rib anomalies as part of the VACTERL association can also be identified. A comprehensive CT reporting checklist (Table 1) describing the important imaging findings should be used.

**TABLE 1:** CT reporting checklist for communicating bronchopulmonary foregut malformations.

S.no.	Feature	Key points
1.	Location	
	Laterality Structure involved	Right/Left Entire lung/lobe/segment
2.	Pulmonary parenchyma	Atelectasis Consolidation Bronchiectasis Air-fluid levels Cystic changes Volume loss Hyperinflation Pulmonary hypoplasia or agenesis
3.	Fistulous communication (detection of anomalous bronchus not arising from the tracheo-bronchial tree)	
	Origin of anomalous bronchus Part of the airway supplied by the anomalous bronchus Course and length of tract Associated tracheal and oesophageal anomalies	Oesophagus (upper or middle or lower) or stomach Entire lung or isolated lung lobe or segment or bronchial communication Patency or calibre Tracheal stenosis or oesophageal atresia or tracheo-oesophageal fistula or gap between blind-ending oesophageal pouch
4.	Vascular anatomy (critical for surgical planning)	
	• Arterial supply	Pulmonary (normal/hypoplastic) Systemic (thoracic or abdominal aorta: origin, course, number and size) Dual supply
	• Venous drainage	Pulmonary veins Anomalous drainage (azygos/hemiazygos system, portal vein, inferior vena cava)
	• Other vascular anomalies	Interrupted pulmonary artery, pulmonary sling, azygous continuation of inferior vena cava
5.	Mediastinum	Heart and major vessels Lymph nodes Mediastinal shift
6.	Complications	Infection Aspiration pneumonia Airway obstruction
7.	Associated anomalies	VACTERL spectrum Bronchopulmonary foregut malformations (duplication cysts, sequestration etc.)

VACTERL, vertebral, anal, cardiac, tracheo-oesophageal, renal and limb.

In summary, both tracheo-oesophageal fistula and CBPFM feature an abnormal patent communication between the foregut and airways. Whereas tracheo-oesophageal fistula is characterised by communication between the oesophagus and trachea, CBPFM demonstrates an abnormal communication between the oesophagus and pulmonary parenchyma by means of an extra or anomalous bronchus, which may supply the entire lung, anatomic lobe or segment of the lung or communicate with the normal bronchial system, with or without additional direct tracheo-oesophageal communication. Radiologists must be aware of the pathogenesis and imaging findings to avoid misdiagnosis of CBPFM as tracheo-oesophageal fistula.^20^

Surgical management of CBPFM involves resection of the affected lung or lung segment, which is usually extensively damaged by recurrent infections. Another alternative is reconstruction of the oesophageal bronchus to salvage the lung; however, the results are not encouraging because of the frequent complication of airway stenosis.^4^

The two presented cases demonstrate the salient imaging features of CBPFM using various modalities. As per the classification, the first case corresponds to group II CBPFM (right lung originating from the lower oesophagus) while the second case, which was a neonate, represents group III CBPFM (isolated left lower lobe communicating with the oesophagus).

## Conclusion

Communicating bronchopulmonary foregut malformations are rare anomalies characterised by an abnormal communication between the lung or lung segment and gastrointestinal tract (oesophagus or stomach). Clinical diagnosis is difficult because the presentation is non-specific and may mimic tracheo-oesophageal fistula. Radiological imaging plays a pivotal role in the diagnosis and management of this condition; contrast oesophagogram and contrast-enhanced CT of the thorax together provide the necessary information for appropriate diagnosis and surgical planning.
